# Comparison of transcriptome technologies in the pathogenic fungus *Aspergillus fumigatus* reveals novel insights into the genome and MpkA dependent gene expression

**DOI:** 10.1186/1471-2164-13-519

**Published:** 2012-10-02

**Authors:** Sebastian Müller, Clara Baldin, Marco Groth, Reinhard Guthke, Olaf Kniemeyer, Axel A Brakhage, Vito Valiante

**Affiliations:** 1 Department of Systems Biology / Bioinformatics, Leibniz Institute for Natural Product Research and Infection Biology - Hans Knöll Institute, Beutenbergstr. 11a, Jena 07745, Germany; 2 Department of Molecular and Applied Microbiology, Leibniz Institute for Natural Product Research and Infection Biology - Hans Knöll Institute, Beutenbergstr. 11a, Jena 07745, Germany; 3 Department of Microbiology and Molecular Biology, Institute of Microbiology, Friedrich Schiller University Jena, Beutenbergstr. 11a, Jena 07745, Germany; 4 Department of Genome Analysis, Leibniz Institute for Age Research - Fritz Lipmann Institute, Beutenbergstr. 11a, Jena 07745, Germany; 5 Integrated Research and Treatment Center, Center for Sepsis Control and Care Jena, University Hospital (CSCC), Jena 07747, Germany

**Keywords:** Aspergillus fumigatus, mRNA-Seq, Transcriptome, Proteome, Secondary metabolite gene clusters, Cell wall integrity pathway

## Abstract

**Background:**

The filamentous fungus *Aspergillus fumigatus* has become the most important airborne fungal pathogen causing life-threatening infections in immuno-compromised patients. Recently developed high-throughput transcriptome and proteome technologies, such as microarrays, RNA deep-sequencing, and LC-MS/MS of peptide mixtures, are of enormous value for systematically investigating pathogenic organisms. In the field of infection biology, one of the priorities is to collect and standardise data, in order to generate datasets that can be used to investigate and compare pathways and gene responses involved in pathogenicity. The “omics” era provides a multitude of inputs that need to be integrated and assessed. We therefore evaluated the potential of paired-end mRNA-Seq for investigating the regulatory role of the central mitogen activated protein kinase (MpkA). This kinase is involved in the cell wall integrity signalling pathway of *A. fumigatus* and essential for maintaining an intact cell wall in response to stress.

**Results:**

The comparison of the transcriptome and proteome of an *A. fumigatus* wild-type strain with an *mpkA* null mutant strain revealed that 70.4% of the genome was found to be expressed and that MpkA plays a significant role in the regulation of many genes involved in cell wall remodelling, oxidative stress and iron starvation response, and secondary metabolite biosynthesis. Moreover, absence of the *mpkA* gene also strongly affects the expression of genes involved in primary metabolism. The data were further processed to evaluate the potential of the mRNA-Seq technique. We comprehensively matched up our data to published transcriptome studies and were able to show an improved data comparability of mRNA-Seq experiments independently of the technique used. Analysis of transcriptome and proteome data revealed only a weak correlation between mRNA and protein abundance.

**Conclusions:**

High-throughput analysis of MpkA-dependent gene expression confirmed many previous findings that this kinase is important for regulating many genes involved in metabolic pathways. Our analysis showed more than 2000 differentially regulated genes. RNA deep-sequencing is less error-prone than established microarray-based technologies. It also provides additional information in *A. fumigatus* studies and as a result is more suitable for the creation of extensive datasets.

## Background

The development of new transcriptome techniques, coupled with the ability of creating and analysing huge databases, has paved the way for system biology’s “Golden Age”. The generation and processing of high-throughput data makes possible the investigation of the genome, transcriptome, proteome and metabolome of living organisms in an overall context. This is particularly useful for gaining a deeper understanding of pathogenic microorganisms [1].

The above holds also true for *Aspergillus fumigatus*, which can be regarded as the most important airborne fungal pathogen. This fungus can cause a life-threatening disease, invasive aspergillosis (IA), in immuno-compromised patients. Patients that suffer from IA still have a low life expectancy. This is due to the lack of reliable diagnostic tools and of efficient antifungal therapies [2,3].

Transcriptome analysis has just recently been applied to *A. fumigatus*. The first genome-sequencing project, applied to the isolate Af293 and coupled with the first transcriptome data available, has been published [4] and a second genome, from the *A. fumigatus* strain A1163, is now available [5]. In the last few years, various microarray platforms have been designed for global transcriptome analyses of *A. fumigatus*. These platforms have all been developed independently of each other using defined sets of primers and probes. However, developing microarray platforms for *A. fumigatus* has already been made outdated by the advent of new technologies based on cDNA sequencing.

To show the potential possibilities of mRNA-Seq to accelerate *Aspergillus* research and to deepen our knowledge about the regulatory function of MpkA, we analysed *A. fumigatus* wild-type and the corresponding mutant lacking the *mpkA* gene by cDNA sequencing and 2D gel-based proteomics. The gene *mpkA* codes for the mitogen activated protein kinase MpkA which acts on the *A. fumigatus* cell wall integrity (CWI) signalling pathway [6,7]. The Δ*mpkA* mutant strain is sensitive to cell-wall active compounds, oxidative stress and heat shock. The function of this gene is also related to polyamine metabolism and to the iron depletion response [8]. Moreover, it affects the expression of several secondary metabolite gene clusters. The Δ*mpkA* mutant strain produces less gliotoxin than the wild type and this is a potent immunosuppressant belonging to the epipolythiodioxopiperazine class of fungal toxins [8,9]. Our transcriptomic and proteomics data also allowed us to compare different omics-techniques. Analysis of the mRNA sequences obtained revealed unexpected novelties in the *A. fumigatus* genome. We also found that 30% of the transcriptionally active *A. fumigatus* genome has not been annotated in the canonical genome databases (*e.g*. CADRE, Broad, and AspGD) [10,11].

In addition, we addressed the question of how the data obtained with these new technologies differ from publically available microarray data. To do this we compared published transcriptome studies carried out by using either microarrays or by applying mRNA-Seq with the mRNA-Seq dataset we generated. This comparison demonstrated that the data varies more among the different microarray platforms than between mRNA-Seq experiments. Additionally, all the collected transcriptome data were compared with proteome data. Although the data obtained by mRNA-Seq was better correlated with the *A. fumigatus* proteome, there was still a relatively low correlation between the two different datasets.

## Results

### mRNA-Seq data set summary

To gain a deeper insight into the regulatory circuits of the MAP-kinase MpkA in *A. fumigatus* we extracted RNA from a null *mpkA* mutant and from the corresponding wild-type strain. We sequenced a total of six libraries, three biological replicates for the Δ*mpkA* strain and three for the wild-type strain. We obtained about 263 million paired end reads. Of these, 193 million (74%) were uniquely mapped against the *A. fumigatus* A1163 genome. Using a stringent RPKM cut-off of 10 reads per gene, 8172 genes (80% of the total annotated genes) were found to be transcribed in the wild-type strain we used. The coverage obtained was similar to that analysed in the dataset published by Gibbons *et al.*[12] for *A. fumigatus* (72% of the genes having an RPKM greater than 10). It was also similar to an mRNA-Seq study in *A. oryzae*[13], where the majority (83.4%) of the genome was covered by at least one read. Requiring coverage of at least 10 overlapping paired-end reads per base-pair, 70.4% (~20 million base pairs) of the genome was found to be expressed. This is remarkable, considering the fact that according to the *A. fumigatus* CADRE genome database, only ~50% of the genome is potentially transcribable. These findings indicated that the *A. fumigatus* transcriptome has been greatly underestimated.

### UTRs prediction and annotation of new genes

**Figure 1:**
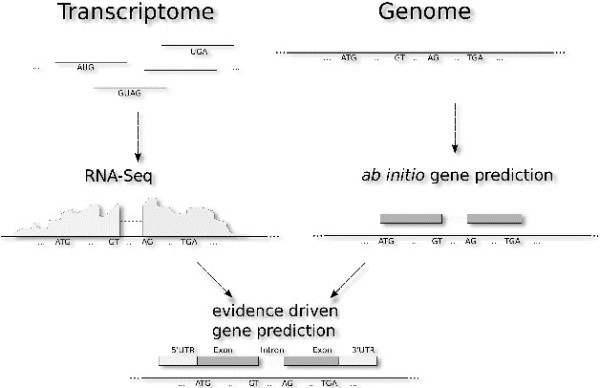
**Workflow for evidence-driven gene prediction. **The evidence is obtained by deep-sequencing which provides valuable hints like splice junctions or expressed regions.

So far, most studies have used mRNA-Seq data to identify transcriptional islands, which are consecutive areas of overlapping reads [13]. We additionally checked these Transcript Active Regions (TARs) for open reading frames. Since this approach is error prone, we employed *ab initio* gene prediction combined with mRNA-Seq data to improve the quality of gene prediction (Figure 1). This evidence-based gene prediction has the advantage over the classic *ab initio* prediction of finding gene structures by making use of the mRNA-Seq expression profiles. This combination incorporates intron-junction information derived from intron-spanning reads and allows the prediction of not translated regions (UTR) in a systematic manner. 

Gene prediction still requires manual work to find appropriate adjustments for integrating data within a classic gene-predicting framework. By using mRNA-Seq data, we identified 185 new transcripts coding for putative proteins ( Additional file 1: Table S1 and Table S2). However, we could not exclude the possibility that the new transcripts are part of untranslated regions (UTRs) or even part of genes that need to be re-annotated. Among all the identified new transcripts, 44 of them have unknown functions, while 141 have orthologs in the genomes of other fungi.

Incorporating mRNA-Seq data into gene prediction also allowed the identification of UTRs ( Additional file 1: Table S2). However, UTR prediction remains a difficult task as only expressed regions provide evidence for UTR length. The method can therefore result in vague length predictions, especially for genes with low expression, due to insufficient coverage. Nevertheless, the identification of 5'UTR can help to find upstream open reading frames (uORFs) and led in our case to the identification of one potential uORF among the newly identified genes ( Additional file 1: Table S2). However, knowledge of UTR sequences in *A. fumigatus* remains poor. This is mainly due to the lack of available expressed sequence tag (EST) libraries. Our analysis highlighted the fact that some genes can have multiple or alternative transcriptional starting points. We found an average median length for 5'UTR of 308 and for 3'UTR of 97 nucleotides based on 9912 UTR sequences for each gene considered ( Additional file 2: Figure S1).

### Comparison of transcriptome technologies

To better evaluate the performance of microarrays and mRNA-Seq, differentially expressed transcripts were investigated. We compared transcript levels in both the wild-type strain and the Δ*mpkA* mutant strain by repeating the experiment performed by Jain *et al.*[8].

**Figure 2:**
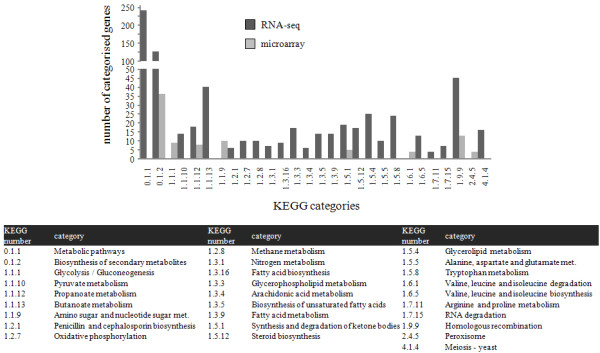
**Comparison of KEGG-enriched categories. **Enriched categories were obtained by analysing two different transcriptome analyses performed by mRNA-Seq (dark grey) and microarray (light grey), both performed to investigate genes expressed differently in a wild type compared to a Δ*mpkA *mutant strain.

Transcriptome data obtained by mRNA-Seq identified 2046 differentially regulated genes (log_2_ fold change >2 and likelihood >0.99). For comparison, using the Febit microarray platform, 653 differentially expressed genes were identified (fold change >1.5) (see Additional file 1: Table S3, Table S4, and Table S5). KEGG analysis showed that common gene categories were enriched by the differentially expressed genes identified in the two datasets (Figure 2 and Additional file 1: Table S6, Table S7, and Table S8). The main difference between data sets was that mRNA-Seq gave a more detailed picture. The number of enriched genes for a specific category was substantially higher showing, for example, complete secondary metabolite gene clusters to be differentially regulated (*e.g*. the gene cluster bordered by genes AFUB_000840 and AFUB_000750). It is noteworthy that genes enriched in the KEGG category 0.1.1., i.e. genes involved in metabolic pathways, were differentially regulated in the mRNA-Seq data, but in contrast, were not enriched in the data set obtained using microarrays. The majority of these genes, in particular genes putatively involved in fatty acid metabolism, were down-regulated in the Δ*mpkA* mutant. This suggests that the RNA deep sequencing technique is more sensitive in finding differentially expressed genes than the microarray technique. This might be explained by the greater dynamic range covered by the RNA technique [14,15]. 

**Figure 3:**
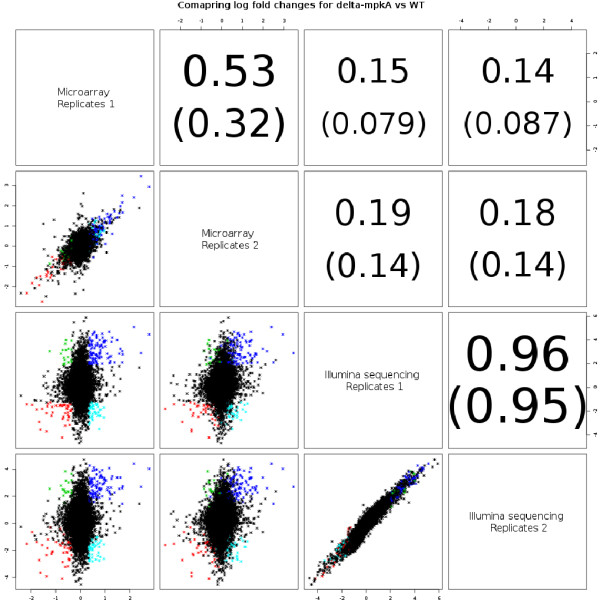
**Comparison of log**_**2**_**fold changes. **Analysis of wt *vs *Δ *mpkA *mutant among and across both technologies (microarrays and mRNA-Seq). Genes are coloured blue if they were up-regulated (being in the 10% quantile of the genes with the highest fold-change) in both technologies (181 DEGs), red if down-regulated in both technologies (141), green if down-regulated on the arrays and up-regulated for sequence platform (70) and cyan for the opposite case (141).

For a more detailed analysis, we computed log fold changes for the Δ*mpkA* mutant *vs* the wild-type of the differentially expressed genes based on both microarray and mRNA-Seq data, including two biological replicates for each (Figure 3). Correlation of log fold changes of expressed genes was highest within the same technology. Correlation within mRNA-Seq replicates (Pearson’s correlation coefficient r = 0.96) was higher than that between the two microarray replicates (r = 0.53). This low correlation was mainly due to genes that only slightly changed their expression between conditions and are therefore more affected by the technological noise resulting from the technology used. A possible explanation is the low sensitivity of microarrays for low expressed genes that results in a smaller dynamic range compared to sequence data [15]. 

When comparing the global transcript fold changes between mRNA-Seq and microarrays the correlation amounted to 0.14-0.19 considering all expressed genes (Figure 3). This was not surprising because most genes are not expected to be differentially expressed, which consequently reduces the correlation. Since these genes are usually not of interest, we selected genes having the highest absolute fold change in both studies (10% quantiles of the total number of genes, intersection from mRNA-Seq and microarrays). The correlation value for this subset resulted in a large increase of both the within-array correlation (from 0.53 to 0.83) and the cross-technology correlation (between mRNA-Seq and microarrays, from 0.19 to 0.41). However, the 0.41 correlation is still lower than those found in other studies (e.g. Marioni *et al*. [16] found a correlation of 0.73 by comparing log fold changes between Affymetrix arrays and Illumina sequencing in experiments performed on human samples).

**Figure 4:**
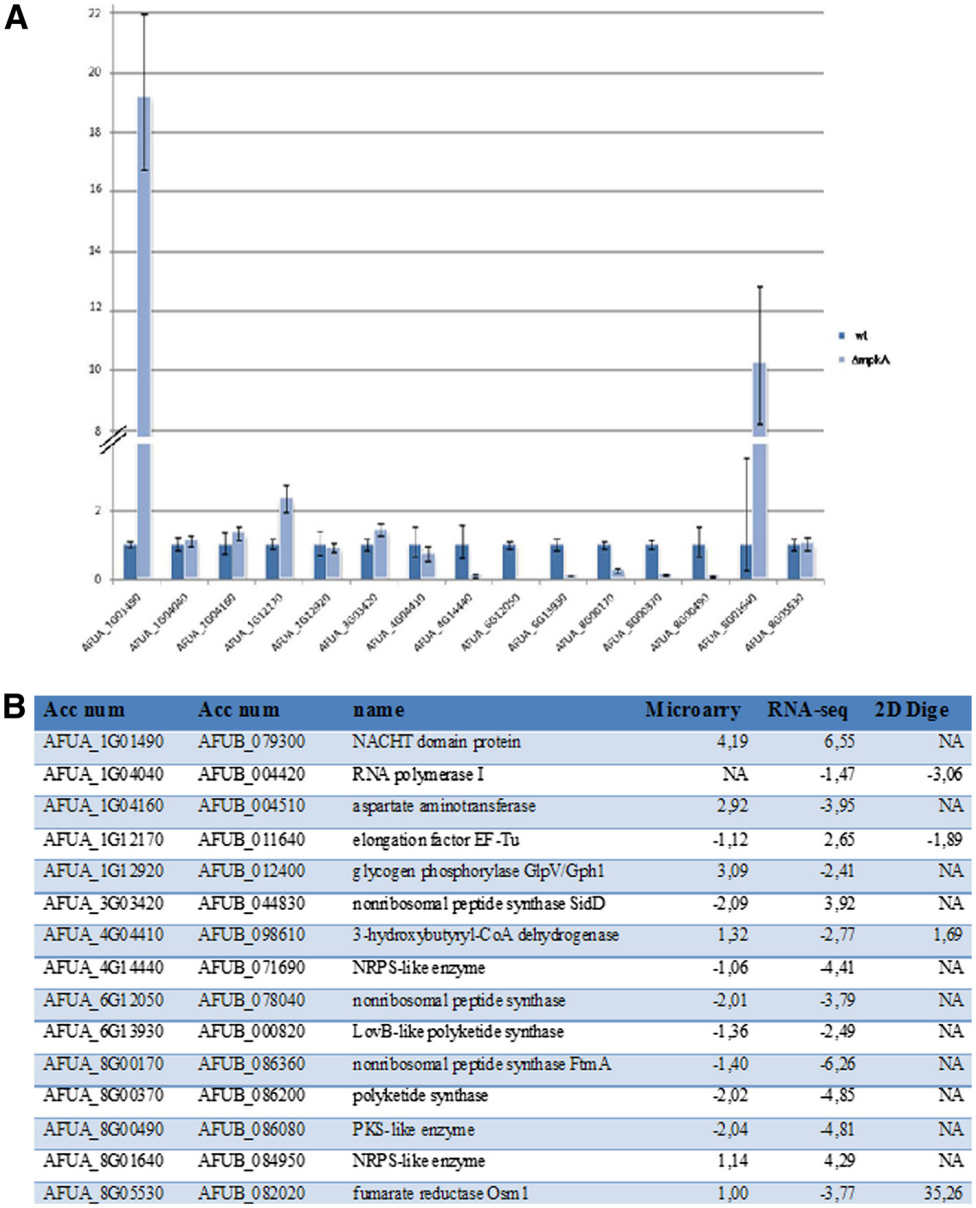
**Differentially expressed gene and protein abundances.A**) qRT-PCR used to check genes that showed differential expression between wt and Δ *mpkA *strains during microarray, mRNA-Seq and 2D proteome analysis. The relative amount of transcripts was normalised by setting the value for each wt gene to 1; **B**) table listing the genes investigated with their respective fold change (Δ *mpkA vs *wt strains) during microarray, mRNA-Seq and 2D proteome analysis.

With the purpose of having additional values for evaluating the transcriptome data from the two technical platforms, qRT-PCR was performed in order to quantify transcript levels of genes that showed different expression levels in both studies (Figure 4, Additional file 1: Table S9 and Additional file 3 :Figure S2). Among the 15 transcripts investigated, 10 confirmed the direction of expression change detected by both transcriptome analysis techniques (with higher significance for mRNA-Seq data). Surprisingly, qRT-PCR did not reveal significant changes in some genes that showed opposite regulation in the analyses of the two techniques. For instance, qRT-PCR confirmed the mRNA-Seq results for the *sidD* gene, coding for a non-ribosomal peptide synthetase involved in siderophore production (AFUA_3G03420). Microarray data showed that genes expressed during low iron stress response were down-regulated in the Δ*mpkA* mutant strain. Previous experiments reversed microarray results, demonstrating that MpkA negatively regulates genes involved in siderophore production, indicating that many genes involved in iron depletion response are up-regulated in the Δ*mpkA* mutant strain (*e.g. sidA* or *sidD*) [8]. Current mRNA-Seq data confirmed our experimental hypothesis, by showing that genes involved in the iron starvation response are, in fact, up-regulated. 

In order to study absolute transcript abundance relationships within and across various microarray and mRNA-Seq platforms, we performed a comprehensive comparison. Analysis of transcriptome techniques included five microarray studies (three different platforms) and two different experiments performed using mRNA-Seq analysis (created in different labs) (Table 1). In this comparison, we included one representative sample corresponding to wild type for each experiment. For the study of Jain *et al.*[8] we also included two independent probe measurements in addition to the standard one (*i.e*., combined) to assess within-platform expression. 

**Table 1:** Arrays analysed

**N**^**o**^	**Technique**	**Platform**	**Used strains**	**Growth conditions**	**Study**	**References**	**Accession**	**Technology**
**1**	Microarray	TIGR (30 k v1)	Af293	5 × 10^6^ conidia per ml, inoculated in complete media, for 17 hours, at 30–37°C, 160 rpm	Temperature shifts (30 to 37°C and 30 to 48°C)	[4]	ArrayExpress: E-MEXP-332	DNA amplicon microarray
**2**	Microarray	Custom (Roche)	Af293	10^8^ conidia per ml, inoculated in potato dextrose media, for 24 hours, at 28°C, 160 rpm	Effects on growth in the presence of TSA	[18]	GEO: GSE19682	60mer oligonucleotide
**3**	Microarray	Febit	CEA17	10^6^ conidia per ml, inoculated in minimal media, for 16 hours, at 37°C, 200 rpm	Comparison among wild type, Δ*gprC*, and Δ*gprD* mutant	[25]	Omnifung	*in situ* oligonucleotide
**4**	Microarray	TIGR (22 K v3)	ATTC 46645	10^7^ conidia per ml, inoculated in Brian’s media, for 14 hours, at 37°C, 150 rpm	Comparison between planktonic and biofilm growth	[17]	GEO: GSE19430	*in situ* oligonucleotide
**5**	Microarray	Febit	CEA17	10^6^ conidia per ml, inoculated in minimal media, for 16 hours, at 37°C, 200 rpm	Comparison between wild type and Δ*mpkA* mutant	[8]	Omnifung	*in situ *oligonucleotide
**6**	mRNA-Seq	Illumina	ATTC 46645 Af293 CEA10*	10^7^ conidia per ml, inoculated in Brian’s media, for 14 hours, at 37°C, 150 rpm	Comparison between planktonic and biofilm growth	[12]	On enquiry	GAIIx
**7**	mRNA-Seq	Illumina	CEA17	10^6^ conidia per ml, inoculated in minimal media, for 16 hours, at 37°C, 200 rpm	Comparison between wild type and Δ*mpkA *mutant	This study	ArrayExpress: E-MTAB-1236	GAIIx
**8**	Proteomic	2D-DIGE	ATTC 46645	10^6^ conidia per ml, inoculated in minimal media, for 16 hours, at 37°C, 200 rpm	Design the *A. fumigatus *mycelial and mitochondrial proteome map	[21]	Omnifung	MALDI-TOF/TOF
**9**	Proteomic	2D-DIGE	CEA17	10^6^ conidia per ml, inoculated in minimal media, for 16 hours, at 37°C, 200 rpm	Comparison between wild type and Δ*mpkA *mutant	This study	On enquiry	MALDI-TOF/TOF

GEO: gene expression omnibus (http://www.ncbi.nlm.nih.gov/geo); Omnifung (http://www.omnifung.hki-jena.de); ArrayExpress (http://www.ebi.ac.uk/arrayexpress/). *CEA17 strain is the uracile auxotroph derived from the CEA10 strain.

**Figure 5:**
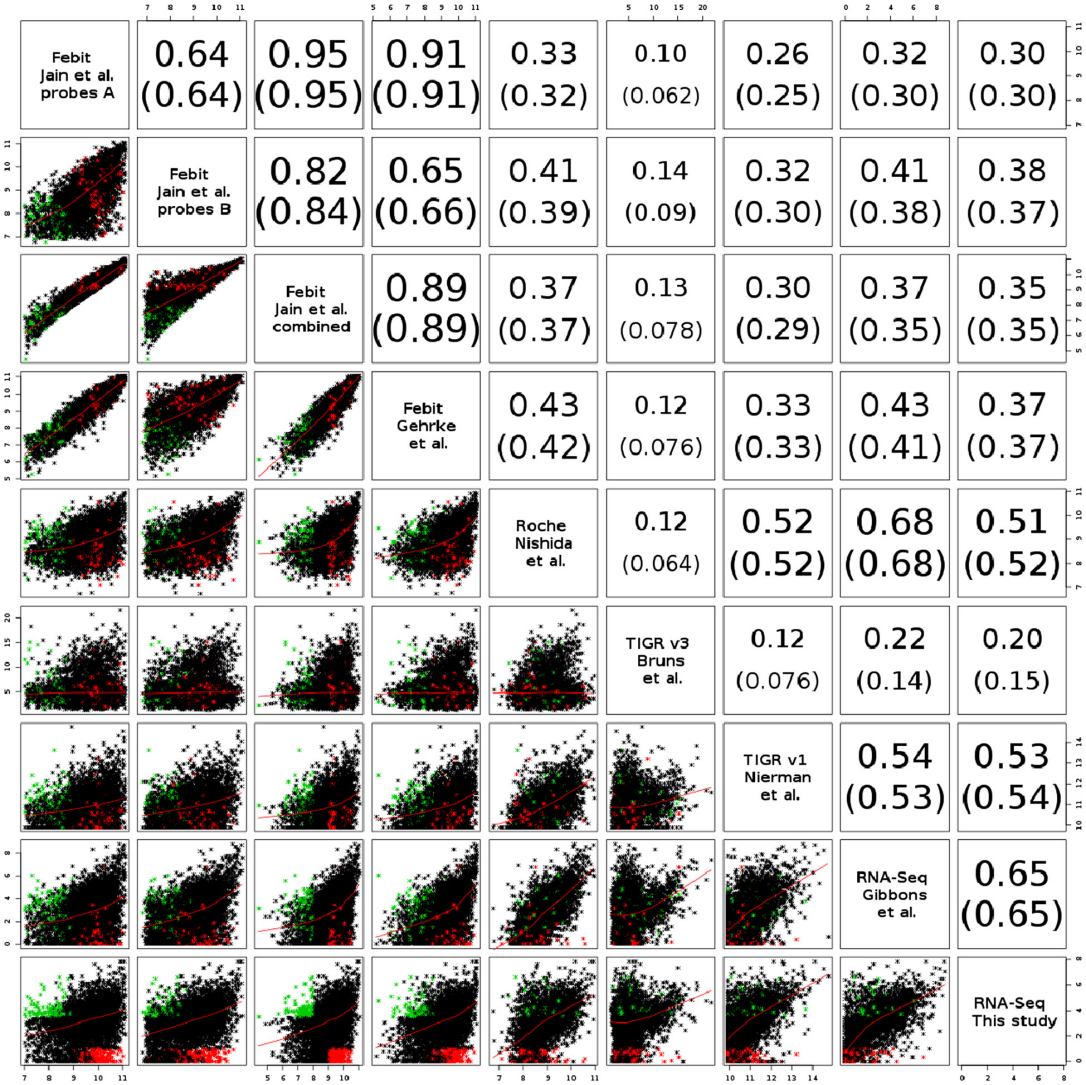
**Pair-wise scatterplot of transcriptome datasets. **Pair-wise scatterplot of the absolute RNA expression values measured by several microarray platforms as well as mRNA-Seq: upper triangle, the pair-wise scatterplots; lower triangle, the corresponding Pearson correlation r (Spearman correlation coefficient r_s_ in brackets) for each pair. A high correlation (with the maximum being one) indicates great agreement of expression between the datasets compared. The green (red) coloured dots correspond to genes that are highly (little) expressed in the mRNA-Seq data and little (highly) expressed in the microarray study of Jain *el al*. (2011) to check for bias due to the different technologies.

In total, the dataset compiled consists of seven wild-type measurements that were compared in a pair-wise manner. A pair wise scatter plot was realised by computing the Pearson and Spearman correlations for all pairs (Figure 5 and Additional file 1: Table S10 and Table S11). Spearman is a rank correlation technique that can capture some non-linear relationships and is less prone to outliers produced by highly abundant genes. As for Marioni *et al*. [16] and Feng *et al*. [14] the correlation between replicates for the same experiment ranged from 0.93 for the dataset of Bruns *et al.*[17] to 0.99 for the both mRNA-Seq studies, indicating high platform-reproducibility for microarrays as well as for sequencing data (data not shown). Therefore, it was sufficient to include only one representative replicate for each platform to obtain concise comparisons. 

The transcriptome data reported showed that expression values measured for transcripts were strongly scattered. Cross platform correlation was quite low, ranging from 0.12 ([17]*vs*[18]) to 0.52 ([4]*vs*[18]). Correlation between the independent measurements (probes A and B) as calculated for the dataset of Jain *et al*. [8] was only 0.64, although all probes were exposed to the same samples. This indicates a strong technological bias of microarray data due to variation in hybridisation. At the same time, the correlation between the two Febit datasets for the combined probe measurements was quite high (0.89) pointing to a high conservation of technological bias in microarray platforms. In order to perform a general comparison between technologies, *i.e*., microarrays *vs* mRNA-Seq, the correlation of data within the same technology was used as a reference for the correlations between technologies. The highest correlation (0.68) among comparisons of mRNA-Seq data to microarray data was higher than the highest correlation between arrays (0.52, Roche vs. TIGR v1). This points to the fact that RNA sequencing data are less affected by technological bias, which is confirmed by the scatter plot comparing both mRNA-Seq datasets (Figure 5).

### Comparison between *A. fumigatus* transcriptome and proteome

In order to analyse a possible correlation between transcripts and protein abundances as well as fold-changes, mRNA-Seq data, microarray data and the proteome data of the *A. fumigatus* wild-type and Δ*mpkA* mutant strain were compared. Looking at differential expression, 48 different proteins were identified by 2D-gel electrophoresis and subsequent MALDI-TOF / TOF, which showed a significant fold change in abundance (employing DIGE) in the Δ*mpkA* mutant compared to wild type. Gene enrichment and pathway analysis revealed that abundance of many proteins involved in metabolic processes and in oxidation/reduction processes is affected by MpkA ( Additional file 1: Table S12 and Additional file 4: Figure S4).

The regulation of protein levels was also compared with transcriptome data obtained from microarrays developed by Febit and used in the study of Jain *et al.*[8] and our mRNA-Seq data. As in earlier results (Figure 3, based on 9800 genes rather than 48) the fold change correlation between microarrays and mRNA-Seq was low (r = 0.14, Additional file 5: Figure S3). The measurements for transcriptome fold change often display low reproducibility because of difficulties in capturing the “true” fold change. For this reason, a low correlation between proteome and transcriptome technologies was assumed. However, the correlation between data obtained by microarrays and the proteome data was significantly higher (r = 0.28) than the correlation between mRNA-Seq data *vs* proteome (r = 0.19). As in the comparative analysis of the transcriptome data, the low correlation applies to all genes with a low fold change resulting in a higher relative impact of technological noise. Furthermore, it has already been shown by studies on human and mouse [19,20] that the relationship between transcriptome and proteome data is quite complex and protein levels are greatly influenced by post-translational processing and inherent variations in stability. These factors result in low correlations.

**Figure 6:**
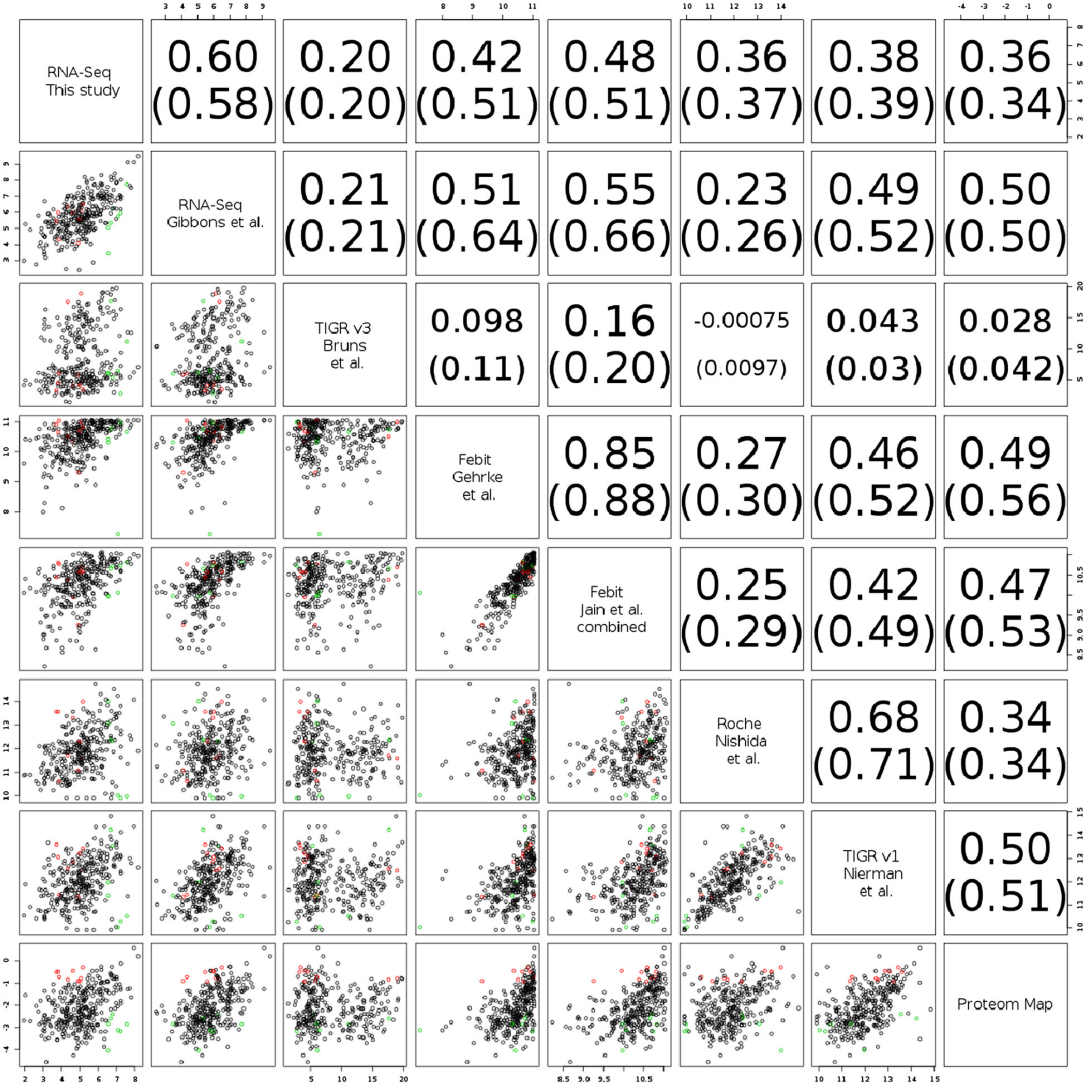
**Pair-wise comparison of all three technologies (microarrays, mRNA-Seq and 2D proteomics). **The last column / row contain the comparison of the proteome-quantities of the proteome map [34] to RNA quantities measured by various microarray as well as mRNA-Seq platforms.

In order to study absolute protein abundances, we reanalysed the *A. fumigatus* 2D proteome map generated by Vödisch *et al.*[21], resulting in the identification of 312 unique proteins. These proteins were also present in the different transcriptome data mentioned above ( Additional file 1: Table S13 and Table S14). In *A. fumigatus*, the relative abundances of the proteome map correlated surprisingly well with mRNA-Seq expression levels (r = 0.36 for our data, and r = 0.5 for data from [12], Figure 5) as well as with most microarrays, in particular the Febit arrays (r = 0.47–0.49). It is also noteworthy that the genes on the scatter plots (Figure 6) are more equally distributed for mRNA-Seq *vs* proteome comparison (left bottom corner) whereas, in particular for Febit arrays, a bias was observed towards genes that are highly expressed on the arrays but with low expression on the proteome-map. This is most probably due to cross-hybridisation. By contrast, this sort of bias was not observed in the NGS data. 

To check for genes that are potentially subject to post-transcriptional regulation, we selected genes that were very abundant in the mRNA-Seq data and rare in the proteome-map (coloured green in Figure 6) or the inverse (coloured red). FunCat-analysis using FungiFun tools [22] indicated that the “green” genes (*i.e.* those genes that are highly expressed according to mRNA-Seq but little expressed according to proteome data) were not significantly enriched within a functional category (even after multiple correction), whereas the red ones (*i.e*. those genes that are little expressed according to mRNA-Seq but highly expressed according to microarray data) were over-represented within the tricarboxylic-acid pathway and amino acid metabolism. These processes constantly require large amounts of proteins that are therefore less subjected to transcriptional regulation.

## Discussion

Over recent years, it has become evident that the eukaryotic regulatory machinery linking cause (for instance, stress) to effect (stress response) is very complex. It usually involves the interplay of several regulatory layers such as transcriptome, epigenome, and proteome that might be subject to independent regulatory mechanisms. To understand these interactions as a whole, integrated studies are required.

We found that about 30% of the *A. fumigatus* genome expressed is located in assigned intergenic and intronic regions. This is most likely due to the presence of untranslated regions (UTRs) and transcripts that have not been classified so far. We investigated the potential of RNA deep-sequencing data to identify these genes by combining gene prediction (using the Augustus platform) and paired-end reads. This pioneer methodology helped us find almost 200 new transcripts with coding potential. Blast analysis revealed that more than half of these new transcripts harbour at least one ortholog in other *Aspergillus* species. This indicates the great potential of this methodology to find new genes and to improve gene annotations.

We tested RNA deep-sequencing to further investigate the role of MpkA in *A. fumigatus*. Previous studies demonstrated that this kinase is decisive for cell wall integrity signalling pathway regulation ([7]; 2010). Further studies revealed that MpkA is also involved in the regulation of the oxidative stress response, secondary metabolite production and siderophore biosynthesis during iron starvation [8]. Concerning the role of MpkA in *A. fumigatus*, mRNA-Seq data confirmed previous findings. Additionally, many genes involved in primary and secondary metabolism were affected by MpkA. In particular, genes putatively involved in amino acid biosynthesis were differentially expressed in the Δ*mpkA* mutant strain. This finding explained the strong difference found in the soluble amino acid pool composition for this strain [8]. Moreover, almost 10% of genes differentially expressed in the Δ*mpkA* mutant are putatively involved in primary metabolism (e.g. fatty acid metabolism and amino acid metabolism). These results confirmed that one of the functions carried out by MpkA is to fine-tune the balance between stress response and energy consuming cellular processes. Considering the genes involved in the iron-deficiency stress response, it was found that different techniques gave opposite results. It was previously reported that MpkA is important for regulating siderophore production by measuring an increased amount of siderophores in the Δ*mpkA* mutant strain during iron starvation [8]. This illustrates that mRNA-Seq is more reliable than other transcriptome techniques. However, the differences found between mRNA-Seq and qRT-PCR showed that it would be good practice if results obtained by sequencing were confirmed by applying *ad hoc* experiments.

Microarray data showed that genes belonging to several *A. fumigatus* secondary metabolite biosynthesis gene clusters were differentially expressed in the Δ*mpkA* mutant (*e.g.* gliotoxin and pseurotin A). Secondary metabolite biosynthesis gene clusters are normally characterised by the presence of polyketide synthases (PKSs) or non-ribosomal peptide synthetases (NRPSs). Analysis of the *A. fumigatus* A1163 genome revealed the presence of 17 NRPSs, 13 PKSs and three hybrid NRPS/PKS enzymes [23].

Among them, 11 gene clusters have been found to be differentially regulated in the Δ*mpkA* mutant. Furthermore, in comparison with the microarray studies, the higher sensitivity of the mRNA-Seq technique led to the identification of almost all genes of a cluster to be differentially regulated. The mRNA-Seq approach is therefore superior to microarrays for studies on the global regulation of secondary metabolite biosynthesis.

The second question that we addressed concerned the comparability of mRNA-Seq data with published transcriptome data. We compared different technologies to investigate the *A. fumigatus* transcriptome (microarrays, mRNA-Seq and qRT-PCR). Information obtained by analysing these technologies were compared with a comprehensive set of proteome data obtained by 2D DIGE analysis followed by MALDI-TOF-MS / MS-based protein detection. The comprehensive dataset compiled in this study constitutes a basis for future studies with the aim of shedding light on the relationship between proteome and transcriptome data, in order, for example, to understand better post-transcriptional regulation on a global scale.

With regard to *A. fumigatus*, the results of whole genome transcriptome analyses based on microarrays were highly dependent on the platform used. Low consistency of microarray gene expression data can have multiple reasons, such as platform effects [24], different media, RNA extraction protocols, or biological variability. However, hybridisation seems to have the most significant impact. Hybridisation strongly depends on the probes, which were used to develop the microarrays. In the case of *A. fumigatus*, they have been designed independently from each other for the different platforms. The hybridisation bias seems to be highly conserved within the same platform, as shown by the high level of agreement between the studies of Jain *et al.*[8] and Gehrke *et al.*[25]. When considering the comparison between Jain *et al.*[8] microarray data with the mRNA-Seq data produced in this study, the correlation is relatively low (r = 0.35). Moreover, comparing the two datasets obtained with the same experimental proceeding by Bruns *et al.*[17] using microarrays and by Gibbons *et al.*[12] using mRNA-Seq, once again showed quite a low correlation (r = 0.22). The global comparison demonstrated that a higher correlation was found between the two mRNA-Seq datasets. This finding demonstrates that mRNA-Seq gives the highest level of comparability in terms of gene expression levels in *A. fumigatus*.

Comparative analysis of the *A. fumigatus* intracellular 2D proteome map from Vödisch *et al.*[21] revealed that protein abundances correlated well with transcriptome abundances measured with both microarray (r = 0.34-0.50) and mRNA-Seq techniques (r = 0.36-0.50). These values were higher than the ones observed by Albrecht *et al.*[26], who compared changes in *A. fumigatus* microarray and proteome data after heat shock induction. Previously, Foss *et al.*[27] calculated a correlation between proteome and transcriptome of r = 0.186 in *Saccharomyces cerevisiae* based on 278 proteins detected by LC-MS / MS analysis. Another study performed by Ghazalpour *et al.*[19] using LC-MS / MS-based quantifications of 486 mouse proteins, resulted in an average correlation of r = 0.27 (using Affymetrix microarray). However, although the results showed a better correlation between *A. fumigatus* proteome and transcriptome data, there was still a relatively low correlation between the two different datasets. This is most likely caused by a combination of several biases based on technological and biological effects.

## Conclusions

The potential of next generation sequencing to investigate the regulation of the *A. fumigatus* transcriptome was demonstrated. We found that the transcriptional potential of *A. fumigatus* was underestimated. Almost 70% of the genome was found to be actively transcribed. Sequence information obtained by mRNA-Seq was also used for gene prediction. We showed that the incorporation of transcriptome-based assembly can be very helpful for improving or confirming gene annotation. This methodology allowed us to identify 185 new transcripts never reported in the *A. fumigatus* A1163 strain. Compared to microarray data, we identified three times more differentially regulated genes in the Δ*mpkA* mutant strain compared with the wild type. Comparative transcriptome and proteome studies pointed out that MpkA plays an important role not only in maintaining the cell wall structure under stress condition but also in affecting genes involved in primary and secondary metabolism.

We compared data obtained by mRNA-Seq with those obtained by established microarray-based technologies for expression profiling. mRNA-Seq was found to be less error-prone and more suitable for the realisation of extensive datasets that can be potentially created by different groups under different lab conditions. Consequently, data produced so far using different microarray platforms, can only be considered by focusing on the highly differentially expressed genes.

## Methods

### cDNA library construction and sequencing

The *A. fumigatus* strain CEA17^KU80^ strain was used for all experiments. The CEA17^KU80^ strain was derived from the CEA17 wild-type strain [28].

Total RNA was isolated from wild-type and Δ*mpkA* strains cultured in *Aspergillus* minimal medium (AMM) for 16 h at 37°C [8], using the Qiagen RNeasy Plant Mini kit (Qiagen, Germany), according to the manufacturer’s instruction. Three biological replicates for each strain were collected. Total RNA was used for Illumina next-generation sequencing [29]. For library preparation 5 μg of total RNA per sample were processed using Illumina mRNA-Seq sample prep kit (RS-100-0801) following the manufacturer’s instruction. The cDNA libraries were sequenced using a Genome Analyser (GAIIx) in a paired-end approach with 2 × 36 cycles resulting in paired reads with a length of 36 nucleotides per read. Each library was sequenced on a single lane and ended up with around 30–40 million reads per sample. Sequence data were extracted in FastQ format and used for further analysis.

### Mapping and normalising of transcriptome reads

All reads were mapped using TopHat 1.2.0 [30] against the *A. fumigatus* A1163 genome (which is a derivative of the *A. fumigatus* CEA17 strain) retrieved from Ensemble release 9. Parameters were set according to preliminary investigations, using a minimum intron length of 30 and a maximum of 4000. In agreement with the protocol used, from preliminary mapping a mean inner distance between mate pairs of 307 was found with a standard deviation of 110. To avoid multiple hits for a single read, the maximum multi-hit option was set to one.

Transcript expression levels were normalised by counting the number of reads per kilobase of exon region per million mapped reads (RPKM) [31].

To detect genes differentially expressed in the wild type and the Δ*mpkA* mutant, we applied the R package baySeq [32] which takes advantage of the three biological replicates for each condition. RPKM and differentially expressed genes were determined using the *A. fumigatus* A1163 genome annotated by CADRE as a reference.

Differentially expressed genes were categorised using GO enrichment, KEGG enrichment, and FunCat analysis using the FungiFun platform [22].

### UTRs prediction

With the purpose of identifying putative new transcripts, we performed new gene annotation employing Augustus *ab initio* prediction [33] combined with hints generated from the mRNA-Seq reads. We present a new annotation including UTRs for *A. fumigatus* A1163. These hints consisted of intron junctions that were identified by TopHat as well as exon-parts revealed with the IRanges package which is part of the Bioconductor collection. The workflow for new gene annotation is reported in Figure 1.

### Microarray data

The transcriptome and proteome data analysed are summarised in Table 1. The datasets were obtained in a pre-processed state *i.e.* background-corrected and normalised. Since the different microarray platforms and even the different *A. fumigatus* genomes have used different Gene-IDs (*e.g.* accession numbers starting with AFUB for the A1163 strain, and accession numbers starting with AFUA for the Af293 strain), all data were mapped according to the AFUA annotation, using ensemble mapping, to achieve comparability. The data were imported and analysed in the R / Bioconductor software environment employing the packages biomaRt and Biostrings.

### Real-Time PCR

The qRT-PCR experiments to determine the amounts of selected putatively differently expressed genes were performed with StepOnePlus Real-Time PCR System (Applied Biosystems), using myTaq HS Mix 2x (Bioline) and Evagreen (Biotium). For each transcript analysed more primers were tested in tandem, in order to obtain primer efficiency close to 100% in all cases. To validate primers, efficiency standard curves were realised considering seven serial dilutions of genomic DNA of *A. fumigatus* in three replicates. All the primers selected for the qRT-PCR analysis are shown in Additional file 1: Table S15. The *A. fumigatus actin1* gene (AFUA_6G04740) was chosen as the normalising gene. The cDNA was generated using RevertAid Premium (Fermentas). As negative controls, a vial was prepared without reverse transcriptase, and another one was prepared without cDNA.

### Assessing differential expression

To correlate the mRNA-Seq data with the corresponding presence of proteins, the mRNA-Seq data set was compared with the proteome data published by Vödisch *et al.*[21]. For our study, the normalised protein spot abundances on the 2D gels were reanalysed using the most recent version of the software Delta 2D 4.3 (Decodon, Germany).

The DIGE (difference in-gel electrophoresis) technique was used to analyse protein samples of wild type and Δ*mpkA* mutant mycelium cultivated in AMM for 16 h at 37°C. Twelve samples (three independent biological replicates and two technical replicates for each strain as well as one mixed internal standard), were labelled with CyDye minimal dyes according to the manufacturer’s protocol with slight modifications (GE Healthcare Bio-Sciences, Munich Germany). We then labelled 50 μg total protein of each sample with 300pmol of CyDye DIGE fluorophores (dissolved in dimethyl formamide). Samples obtained from the wild-type and from the Δ*mpkA* mutant strain were labelled with either Cy3 or Cy5. A pool of all six samples (3 × wt and 3 × Δ*mpkA*) was prepared, labelled with Cy2, and used as a global internal standard. We carried out 2D gel processing, spot screening and MALDI-TOF / TOF analysis with an ultrafleXtreme mass spectrometer (Bruker Daltonics, Germany), as previously described [34] with slight modifications. FlexControl 3.3 software was used for data collection and flexAnalysis 3.3 for spectra analysis and peak list generation (Bruker Daltonics, Germany). Peptide mass fingerprint (PMF) and peptide fragmentation fingerprint (PFF) spectra were submitted to the MASCOT 2.3 server (Matrix Science, U.K.) searching the recent version of the NCPInr database but restricted to the fungi. Mascot search parameters were the following: fixed modification of cysteine thiols to S-carbamidomethyl derivatives, variable methionine oxidation, up to one missed cleavage and a peptide mass tolerance of 100 ppm.

### Accession number

The RNA-Seq data were deposited in ArrayExpress (ArrayExpress accession: E-MTAB-1236).

## Competing interests

The authors declare that they have no competing interests.

## Authors’ contributions

VV and SM designed research. SM, MG, CB and OK performed research. SM, RG, OK, AAB, and VV analysed data and wrote the paper. All authors read and approved the final manuscript.

## Supplementary Material

Additional file 1 Tables.xls (Excel file which includes all the cited supplementary tables). Click here for file

Additional file 2 **Figure S1.** Length distribution of untranslated regions (UTRs). The analysis was based on 9912 3' and 5' UTR sequences. The red vertical lines indicate the average median length of the 5'UTRs (308) and the 3'UTRs. Click here for file

Additional file 3 **Figure S2. **Comparison of delta_delta_Ct values obtained by qRT-PCR analysis (see also Additional file 1: Table S9). Values obtained by qRT-PCR analysis where compared to log_2_ fold changes (wt vs Δ*mpkA*) obtained by microarrays and mRNA-Seq based on 14 genes. Both technologies seem to agree with the qRT-PCR-data with an average Pearson correlation of r = 0.75 for microarrays and 0.55 for mRNA-Seq (Spearman correlation coefficient r_s_ in brackets). Click here for file

Additional file 4 **Figure S4. **2D gel electrophoresis of protein extracts. Total proteins were extracted from *A. fumigatus *wild type (Cy5, green) and Δ*mpkA * mutant strain (Cy3, purple). Proteins were stained with the difference in gel electrophoresis (DIGE) labelling technique. Total proteins were separated in a pH gradient of 3–11 (nonlinear). Click here for file

Additional file 5 **Figure S3. **Pair-wise scatterplot of all three technologies. Comparison of three different technologies that is 2D-DIGE proteomic, mRNA-Seq and microarray, to detect differentially expressed genes and proteins between the Δ*mpka *strain and wild-type strain, based on log fold changes of 94 entries. The overall correlation was found to be low, especially between mRNA-Seq and proteomic, whereas the correlation between proteomic and microarray was higher. Click here for file
