# Supplementary figures and images for: Comparison of transcriptome technologies in the pathogenic fungus Aspergillus fumigatus reveals novel insights into the genome and MpkA dependent gene expression
Source: BMC Genomics. 2012 Oct 2;13:519. doi: 10.1186/1471-2164-13-519 (PMC3505472; doi:10.1186/1471-2164-13-519)

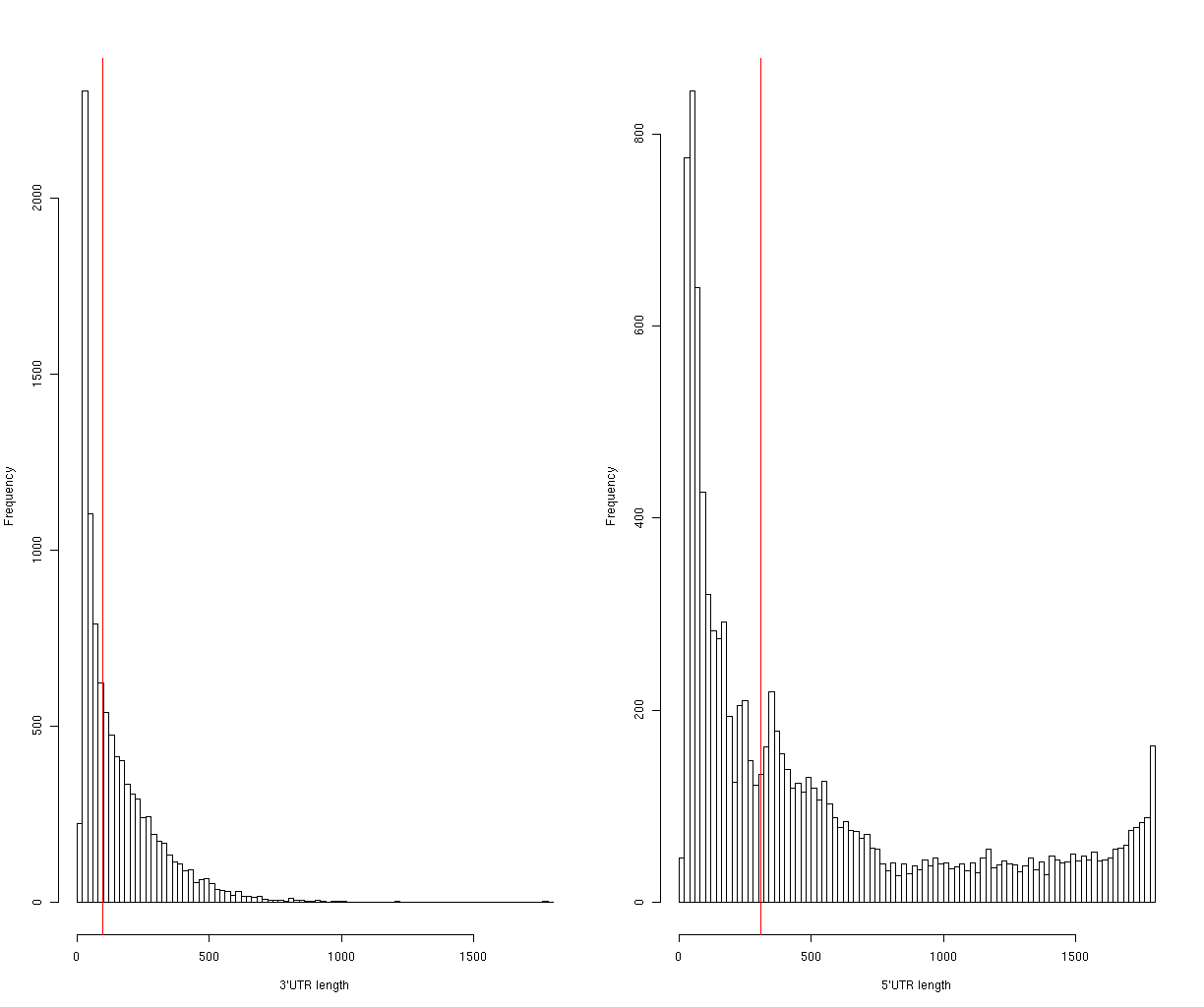

Supplement: Additional file 2 — Figure S1. Length distribution of untranslated regions (UTRs). The analysis was based on 9912 3' and 5' UTR sequences. The red vertical lines indicate the average median length of the 5'UTRs (308) and the 3'UTRs. [file 1471-2164-13-519-S2.png]

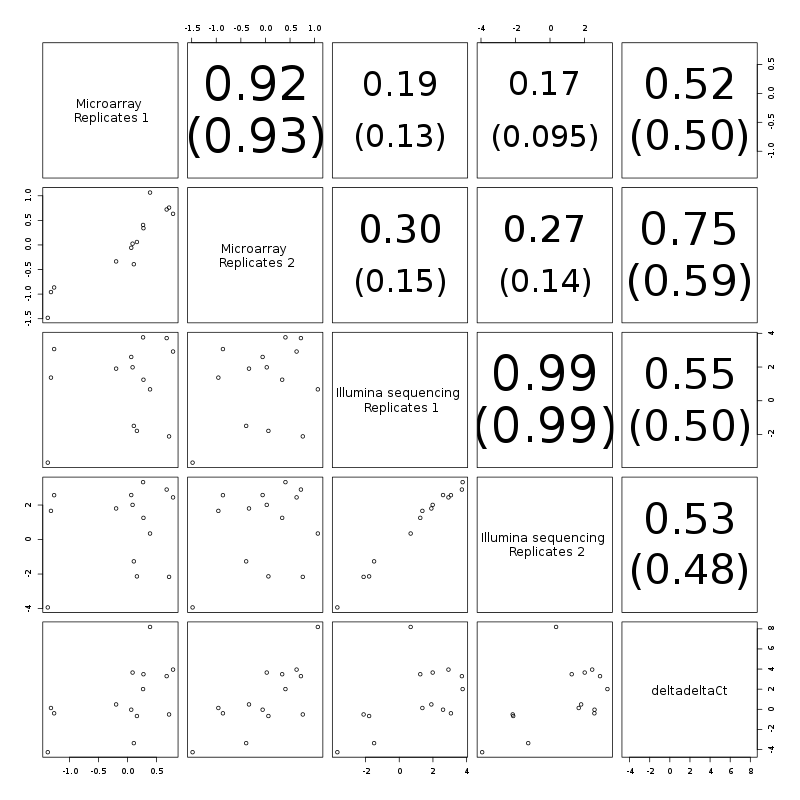

Supplement: Additional file 3 — Figure S2. Comparison of delta_delta_Ct values obtained by qRT-PCR analysis (see also Additional file 1: Table S9). Values obtained by qRT-PCR analysis where compared to log2 fold changes (wt vs ΔmpkA) obtained by microarrays and mRNA-Seq based on 14 genes. Both technologies seem to agree with the qRT-PCR-data with an average Pearson correlation of r = 0.75 for microarrays and 0.55 for mRNA-Seq (Spearman correlation coefficient rs in brackets). [file 1471-2164-13-519-S3.png]

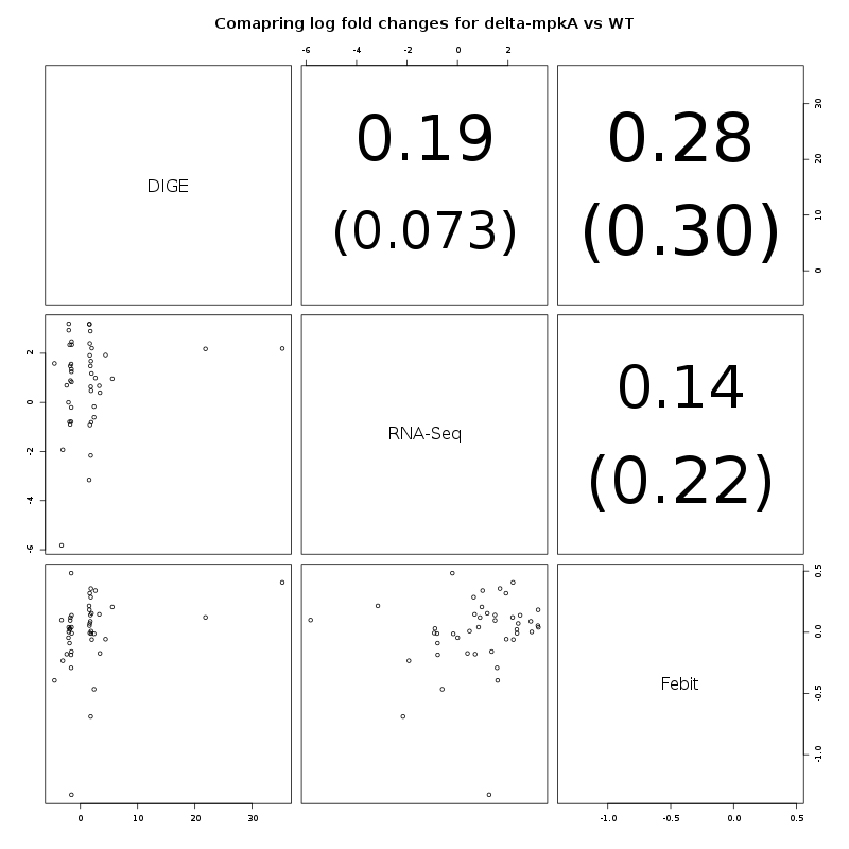

Supplement: Additional file 5 — Figure S3. Pair-wise scatterplot of all three technologies. Comparison of three different technologies that is 2D-DIGE proteomic, mRNA-Seq and microarray, to detect differentially expressed genes and proteins between the Δmpka strain and wild-type strain, based on log fold changes of 94 entries. The overall correlation was found to be low, especially between mRNA-Seq and proteomic, whereas the correlation between proteomic and microarray was higher. [file 1471-2164-13-519-S5.tiff]
